# Synovial distribution of “systemically” administered acetylsalicylic acid in the isolated perfused equine distal limb

**DOI:** 10.1186/1746-6148-9-56

**Published:** 2013-03-26

**Authors:** Maren Friebe, Stephan Schumacher, Jessica Stahl, Manfred Kietzmann

**Affiliations:** 1 Department of Pharmacology, Toxicology and Pharmacy, University of Veterinary Medicine Hannover, Foundation, Bünteweg 17, Hannover, 30559, Germany

**Keywords:** Acetylsalicylic acid, Salicylic acid, Isolated perfused equine distal limb, Synovial fluid, Horse, Microdialysis

## Abstract

**Background:**

This study investigated synovial concentrations of acetylsalicylic acid (ASA) and its metabolite salicylic acid (SA) in the equine fetlock joint following systemic administration of ASA. Salicylates were chosen because SA is the only nonsteroidal anti-inflammatory drug for which threshold levels exist for plasma and urine in equine sports. To avoid animal experiments, the study was conducted using an *ex vivo* model of the isolated perfused equine distal limb in combination with plasma concentrations obtained from literature.

Salicylate concentrations in the joint were determined using microdialysis and high performance liquid chromatography (HPLC). Any anti-inflammatory effect of synovial ASA concentrations was assessed using an ASA EC_50_ (half maximal effective concentration) determined in equine whole blood.

**Results:**

The ASA concentration in the synovial fluid (n = 6) reached a maximum of 4 μg/mL, the mean concentration over the entire perfusion period was 2 μg/mL. Maximum SA concentration was 17 μg/mL, the average was 14 μg/mL. ASA and SA concentration in the synovial fluid exceeded systemic concentrations 2 h and 3.5 h after “systemic” administration, respectively.

**Conclusions:**

ASA and SA accumulated in the in the synovial fluid of the *ex vivo* model despite decreasing systemic concentrations. This suggests a prolonged anti-inflammatory effect within the joint that remains to be further elucidated.

## Background

Acetylsalicylic acid (ASA) belongs to the group of nonsteroidal anti-inflammatory drugs (NSAIDs) and is rapidly converted to its metabolite salicylic acid (SA) after administration. In horses, ASA is not routinely used for the treatment of inflammatory conditions because of its short half-life, low potency and resulting high dosages needed [1-3]. Still, ASA and SA pharmacokinetics are of interest because SA is the only NSAID with threshold levels for plasma and urine in equine sports [4-6]. These threshold levels have been introduced to avoid positive cases due to salicylates originating from plants potentially present in equine feed, e.g. in alfalfa hay [7]. However, threshold levels might increase the risk of ASA being abused for therapeutic purposes up to the threshold in competition horses [8]. In this context, we were interested in synovial fluid concentrations after systemic administration because none of the numerous studies that have dealt with salicylate pharmacokinetics in humans [9-11], cattle [12], and horses [1,3] has so far explored the distribution of ASA or SA into joint fluid. Therefore, the study presented aimed to investigate to what extent ASA is distributed into the synovial fluid of horses after systemic administration.

To avoid ethical limitations arising from the use of study horses and to minimize costs, an *ex vivo* model of the isolated perfused equine distal limb recently established in our group was used [13]. Imitating systemic application of ASA in this model was possible because plasma concentrations of ASA and SA were readily available from an *in vivo* study investigating ASA pharmacokinetics in horses [1].

In the synovia of the *ex vivo* model, salicylate concentrations were monitored using the microdialysis technique. This involves placement of a tubular microdialysis probe into the synovial cavity: the probe comprises a semi-permeable membrane and is constantly perfused with a physiological buffer solution, the so-called perfusate, at a low flow rate. Unbound drug molecules present in the synovial fluid will diffuse through the semi-permeable membrane into the perfusate, down their concentration gradient. Larger molecules, such as proteins and enzymes, cannot cross the membrane. However, an equilibrium between the perfusate and the periprobe fluid will never be reached because of the perfusate’s constant movement. The concentration of the analyte in the perfusate will therefore always be less than in the periprobe fluid. The ratio between these concentrations is defined as relative recovery (RR) and needs to be determined for each probe *in vitro* in order to correctly interpret concentrations measured in the perfusate. The microdialysis technique has already been applied for sampling of SA in skin-penetration studies [14-16].

To get some orientation concerning the effect of plasma protein binding on the synovial distribution of ASA and SA, the experiment was repeated with autologous, diluted blood as perfusate, referred to as hemoperfusion experiments throughout this paper.

## Methods

### Isolated perfused equine distal limb

#### Tyrode perfusion

Six limbs from six different horses were used for this study; each limb was used for one experiment. The preparation of the limb model was performed as described previously [13]. In brief, distal limbs of warmblood horses of various sex and age were obtained from a slaughterhouse where they were exarticulated in the middle carpal joint within a maximum of 30 min after slaughter. Immediately afterwards, the median artery was canulated and infused with heparinised (120 IU/mL; Euro OTC Pharma GmbH, Bönen, Germany) tyrode solution (136.8 mmol/L NaCl, 2.7 mmol/L KCl, 1.8 mmol/L CaCl_2_ × 2H_2_O, 1.05 mmol/L MgCl_2_ × 6H_2_O, 0.416 mmol/L NaH_2_PO_4_ × 2H_2_O, 11.9 mmol/L NaHCO_3_ and 5.5 mmol/L D(+)-glucose × 1H_2_O) to prevent clot formation in the vessels. Extremities were transported to the laboratory and perfused with oxygenated (carbogen: 95% oxygen and 5% carbon dioxide) tyrode solution containing sodium carboxymethyl cellulose (0.15 g/L, Caesar & Loretz, Hilden, Germany) to increase the oncotic pressure in the vessels and therefore counteract edema formation. Perfusion occurred via the median artery over a period of 8 h, during which the flow rate of approximately 65 mL/min was upheld by a peristaltic pump (Masterflex 7553–76; Cole-Palmer Instruments Co, Vernon Hills, IL, USA). The temperature of the perfusion fluid was maintained at 37°C by a water bath. Perfusion started within 60 min after slaughter.

Tissue viability was assessed for each extremity throughout the perfusion period as follows: perfusate samples were taken from the radial vein after 4 h and 8 h of perfusion to determine glucose utilization, lactate production, and lactate dehydrogenase (LDH) release. Furthermore, the skin surface temperature was measured in hourly intervals. The extremity’s weight was recorded before and after the experiment to assess edema formation. Based on results published earlier [13], the following requirements for tissue viability were to be fulfilled by isolated perfused limbs in order to be regarded as viable and therefore evaluable:

1. Glucose utilization ≥ 200 mg/h

2. Lactate production ≤ 400 mg/h

3. LDH activity ≤ 10 U/h

4. Skin surface temperature ≥ 26°C

#### Hemoperfusion

After conclusion of the tyrode perfusion experiments, two extremities of different horses were perfused with diluted autologous blood in order to give a rough overview of the influence of plasma protein binding on the synovial distribution of ASA and SA. The duration of perfusion was reduced to 5 h because this was the period in which the main concentration changes had occurred in the precedent tyrode perfusion experiments. For hemoperfusion, extremities were prepared as described above. In addition, 10 L of autologous blood were collected during exsanguination; anticoagulation was achieved with heparin (18 IU/mL). After transport to the laboratory, the extremity was connected to the perfusion system via the median artery and perfused with tyrode solution for 30 min to remove all remaining blood in the vessels. In accordance with Bäumer et al. [17], the leg was subsequently perfused with a blood-tyrode mixture (4:1) referred to as hemoperfusion perfusate in an open perfusion system. The blood was oxygenated by means of the counter-current-principle with a dialyser (Diacap® hollow fiber dialyser, Braun AG, Melsungen, Germany), through which the hemoperfusion perfusate and 8 L of tyrode solution gassed with pure oxygen were pumped in opposite directions. The tyrode solution in the dialysis circle was continuously moved by a rotary pump (EHEIM, Deizisau, Germany) at a speed of approximately 0.5 L/min. The hemoperfusion perfusate was warmed to 37°C by a water bath and transported to the limb by a peristaltic pump at approximately 65 mL/min.

For determination of tissue viability, two samples of the hemoperfusion fluid were taken in hourly intervals over the entire perfusion period of 5 h: One “arterial” sample from the median artery and one “venous” sample from the site of venous outflow. Samples were centrifuged at 800 g for 10 min and plasma stored frozen at - 20°C until analysis for glucose, lactate and lactate-dehydrogenase concentrations. Glucose consumption in the tissue was calculated by subtraction of glucose concentrations in the venous from those determined in the arterial samples. For lactate production and LDH activity, values in the arterial were subtracted from those in the venous samples. Viability criteria were the same as for the tyrode-perfused limbs.

#### Addition of acetylsalicylic and salicylic acid to the perfusion fluid

To simulate the systemic administration of ASA (20 mg/kg bodyweight) to the isolated perfused equine distal limb, plasma concentrations of ASA and SA from a study investigating plasma concentrations after intravenous administration in horses [1] were added to the perfusion medium. *In vivo*, ASA would rapidly be converted to SA via deacetylation by tissue and plasma esterases. Since the *ex vivo* model lacks these mechanisms, the respective ASA/SA ratio was imitated in the perfusion medium as indicated in Table 1. Salicylate concentrations in the perfusion fluid were adjusted to the respective *in vivo* plasma concentrations in 30-min-intervals during the first hour and in 60-min-intervals until the end of the experiment. Hence, during the first 30 min after virtual systemic administration of ASA, the perfusion medium contained 100 μg/mL ASA and 50 μg/mL SA (both Sigma Aldrich, Steinheim, Germany). After 30 min, the perfusion medium was exchanged so that it contained 40 μg/mL ASA and 125 μg/mL SA and so forth. Actual ASA and SA concentrations in the perfusion medium were verified by HPLC analysis.

**Table 1:** Simulated intravenous administration of ASA (20 mg/kg bodyweight) in the isolated perfused equine distal limb according to Broome et al., 2003

**Time (h)**	**0 - 0.5**	**0.5 - 1**	**1 - 2**	**2 - 3**	**3 - 4**	**4 - 5**	**5 - 6**	**6 - 7**
***ASA***								
μg/mL	100	40	5	0	0	0	0	0
***SA***								
μg/mL	50	125	70	35	20	5	5	5

ASA and SA were added to the perfusion fluid in the respective concentrations indicated below; microdialysis sampling vials were exchanged with every concentration adjustment.

The time point of simulated systemic intravenous application was 1 h after the start of perfusion, because the first 30 min of perfusion were allowed for equilibration and another 30 min were needed to collect a blank sample after implantation of the microdialysis probe.

### Microdialysis

#### Determination of relative recovery *in vitro*

Prior to measuring synovial salicylate concentrations in the *ex vivo* model, the relative recovery (RR) of ASA and SA in equine synovial fluid was established *in vitro* for all six microdialysis probes in order to ensure reproducible drug sampling. Concentric probes of 0.5 mm membrane diameter, 20 kD molecular weight cut-off and a membrane length of 10 mm were used (CMA 20, CMA Microdialysis AB, Stockholm, Sweden). For calibration, the entire probe membrane was placed in a 0.5 mL reaction tube with freshly prepared equine synovial fluid containing 1, 5, 10, or 15 μg/mL ASA or SA. These concentrations had been determined in preliminary experiments (data not shown) in order to obtain an adequate calibration range.

The microdialysis probe was perfused with phosphate buffered saline (8 g/L NaCl, 0.2 g/L KCL, 0.2 g/L KH_2_PO_5_, 1.44 g/L Na_2_HPO_4_ x 2 H_2_0) at a flow rate of 3 μL/min using a TSE 540063 syringe pump (TSE Systems, Bad Homburg, Germany). For each concentration, the dialysate was collected for 60 min following a 10-minute equilibration period. Experiments were performed at room temperature and repeated on three consecutive days. The procedure was performed for ASA and SA. Respective ASA and SA concentrations in the dialysate were determined with HPLC. Relative recovery was calculated for each probe and concentration by dividing the concentration in the dialysate C_Dialysate_ by the concentration in the synovial fluid C_Synovia_ (equation 1).

(1) $${\mathrm{RR}=C}_{\mathrm{Dialysate}}/C_{\mathrm{Synovia}\;\mathrm{in}\;\mathrm{vitro}}$$

The average relative recovery for ASA and SA was calculated for each probe in order to determine the respective salicylate concentration in the joint of the *ex vivo* model in the following experiments (equation 2).

(2) $$C_{\mathrm{Synovia}\;\mathrm{ex}\;\mathrm{vivo}}=C_{\mathrm{Dialysate}}/\mathrm{RR}$$

#### Microdialysis in the isolated perfused equine distal limb

After an equilibration period of 30 min, a calibrated microdialysis probe was implanted into the fetlock joint of the isolated perfused equine distal limb via the dorsal pouch as follows: a stab incision was made through the skin where probe implantation was planned. A steel needle was inserted into a split tubing (“introducer”) and introduced into the dorsal pouch of the fetlock joint. Placement of the needle in the joint was confirmed by aspiration of synovial fluid. The steel needle was then removed from the introducer and the microdialysis probe inserted instead. In a last step, the flexible introducer was pulled out of the joint by splitting it around the microdialysis probe. The probe was perfused with PBS at a flow rate of 3 μL/min and sampling vials exchanged as indicated in Table 1. Samples were frozen at −20°C immediately after collection until analysis. One probe was used for each extremity; two probes were used a second time for the hemoperfusion experiments.

### Analytical method and analysis

ASA and SA concentrations were measured in the microdialysis perfusate using a validated HPLC method with limits of detection at 53 ng/mL and 84 ng/mL and limits of quantification at 106 ng/mL and 169 ng/mL for ASA and SA, respectively. Validation of the method included evaluation of the following characteristics: accuracy and precision, linearity, and specificity/selectivity. Validation experiments followed the FDA Guidance for Bioanalytical Method Validation [18]; all results were in the range of the recommended limits.

The microdialysis perfusate could be readily analysed without extraction; 100 μl of each sample were injected into the isocratic HPLC system (Beckman Coulter GmbH, Krefeld, Germany) comprising a 126 solvent module, a 508 autosampler and a 166 Detector. The column used was a LiChroCART® 125–4 LiChrospher® 100 RP-18, 5 μm column coupled with a guard column (LiChroCART® 4–4; LiChrospher® 100 RP-18, 5 μm; both Merck KGaA, Darmstadt, Germany). Columns were kept in a column oven (SpH 99, Spark Holland, Emmen, Netherlands) at 40°C. Instrument control and data collection were performed with the software 32 Karat (Beckman Coulter GmbH, Krefeld, Germany). The mobile phase consisted of HPLC-grade acetonitrile (Lab-Scan, Gliwice, Poland) and McIlvaine citrate buffer pH 2.2 (40:60, v/v); flow rate was 1 mL/min and UV detection occurred at 310 nm.

### Statistical analysis

Data are expressed as mean ± S.E.M. of independent experiments as specified in individual figure legends. All graphs were created by GraphPad Prism 5.03 software (GraphPad Software, Inc., La Jolla, CA, USA).

## Results

### Tissue viability

Tissue viability requirements were met by all 8 perfused distal limbs (6 tyrode-perfused and 2 hemoperfused limbs). The average weight increase was 5.1% after 8 h of perfusion with tyrode and 8.6% after 5 h of hemoperfusion.

### Determination of relative recovery *in vitro*

Mean values for relative recovery are shown in Table 2; mean recovery in all probes was 25.2 ± 2.2% for ASA and 9.1 ± 1.1% for SA (mean ± S.E.M., n = 6).

**Table 2:** **Relative recovery (RR) of ASA and SA in equine synovia for microdialysis probes (CMA 20, CMA Microdialysis AB, Stockholm, Sweden) in calibration experiments *in vitro***

***Probe #***	***RR ASA%***	***RR SA%***
1	26.5	13.8
2	35.5	7.7
3	23.7	6.1
4	20.9	10.7
5	20.6	7.1
6	23.9	9.2
**Mean**	**25.2**	**9.1**
**S.E.M.**	**2.2**	**1.1**

Data are given as mean of 4 different concentrations (1, 5, 10 and 15 μg/mL) repeatedly measured on 3 consecutive days.

### *Ex vivo* microdialysis in the isolated perfused equine distal limb - Tyrode

**Figure 1:**
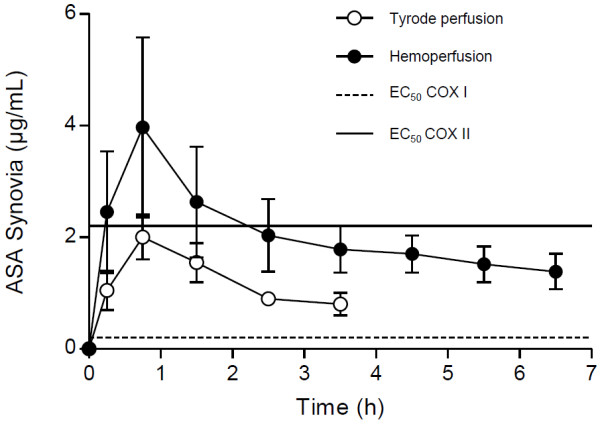
**ASA concentrations in the synovial fluid of the fetlock joint in the isolated perfused equine distal limb after imitated systemic administration of 20 mg/kg ASA.** Synovial fluid concentrations were measured using microdialysis and HPLC and are given as mean ± S.E.M of 6 (tyrode perfusion) or 2 (hemoperfusion) independent experiments. ASA EC_50_ values for COX I and COX II (Buntenkötter, 2012) are indicated to assess the anti-inflammatory effect of the synovial concentrations.

**Figure 2:**
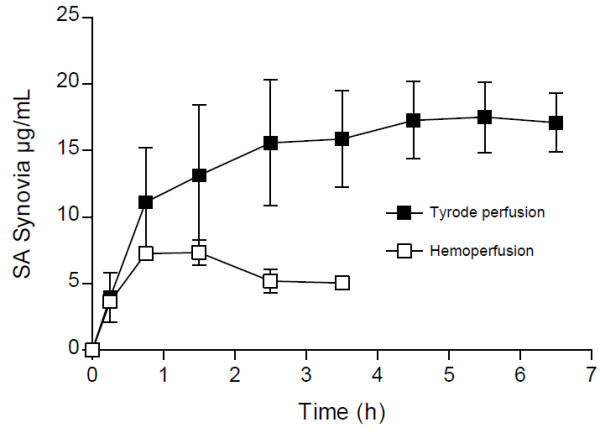
**SA concentrations in the synovial fluid of the fetlock joint in the isolated perfused equine distal limb after imitated systemic administration of 20 mg/kg ASA.** Synovial fluid concentrations are given as mean of 2 independent experiments (hemoperfusion) and mean ± S.E.M. of 6 independent experiments (tyrode perfusion).

ASA and SA were detectable in all 6 experiments, synovial fluid concentrations in the fetlock joint are shown in Figures 1 and 2. ASA reached the highest synovial concentration of 4 μg/mL shortly after application before decreasing to approximately 2 μg/mL until the end of the experiment. Mean ASA concentration in the synovial fluid over the entire perfusion period was 2 μg/mL. The drug was detectable until the end of each experiment even though its supply via the perfusion medium ceased 2 h after “systemic” application, reflecting conditions *in vivo*.

During the first 5 h after “treatment”, SA concentrations continuously increased to reach a plateau and maximum concentration at 17 μg/mL. Mean SA concentration in the synovial fluid throughout the entire experiments was 14 μg/mL. None of the blank samples taken before onset of drug administration contained any measurable amount of salicylates.

### *Ex vivo* microdialysis in the isolated perfused equine distal limb – Hemoperfusion

In the hemoperfused extremities (n = 2), ASA and SA were detectable in the synovial fluid of the fetlock joint until the end of the experiment after 4 h (Figures 1 and 2). Mean ASA concentration over the entire perfusion period was 1.1 μg/mL, synovial concentrations decreased from the initial maximum concentration of 2 μg/mL to 0.9 μg/mL 3 h after “systemic” administration. SA concentration showed an initial increase with a subsequent decline and ranged from 4 to 7.3 μg/mL, the average being 5.7 μg/mL. Concentrations of ASA and SA supplied via the hemoperfusion fluid are displayed in Table 1. Again, no salicylates could be detected in any of the blank samples taken before drug administration.

## Discussion

### *Ex vivo* model

For each limb, tissue viability was ensured by the criteria described under the methods section. While establishing the model, special attention was paid to the integrity of the synovial membrane [13]. We therefore assumed the distribution of salicylates into the synovial cavity in the *ex vivo* model to be comparable to the *in vivo* situation.

To get a rough overview of the role of plasma protein binding on the synovial distribution of ASA and SA, perfusion was performed with diluted autologous blood (mixed with plasma in a ratio of 1:4) in two extremities. Comparison of the mean synovial fluid concentrations in the tyrode- and hemoperfused limbs revealed ASA and SA concentrations to be approximately 50% of the synovial fluid concentrations in tyrode-perfused limbs. This difference might be due to the plasma protein binding of salicylates which is reported to be around 50% [19].

However, pathological changes affecting the permeability of the inner joint capsule are known to alter the rate of transport of salicylates in man: it has been suggested that e.g. hyperemia and edema formation due to synovitis in acute osteoarthritis might accelerate salicylate transport from the bloodstream to the joint, whereas hyperplastic thickening of the capsule or the synovial vessels in conditions such as chronic osteoarthritis might hinder this transport [20]. The possible influence of these conditions could not be examined in this study because so far, the model has been established to mirror conditions only in the healthy limb.

### Distribution of salicylates into synovial fluid

In the study presented, the distribution of salicylates into joint fluid after systemic administration was measured. Although not frequently used in equine medicine, these drugs are of peculiar interest because of their special status as threshold substances.

The *ex vivo* model of the isolated perfused equine distal limb was used to avoid animal experiments. In order to imitate systemic administration of ASA, salicylate plasma concentrations in the perfusion fluid of the *ex vivo* model were adjusted in accordance with plasma concentrations reported in an *in vivo* study. Synovial concentrations of ASA and SA were monitored in the fetlock joint of the model using microdialysis.

Even though it has been stated that the transport of the unbound fraction of a drug through a synovial membrane is a simple diffusion process [21,22], data obtained from our experiments suggest an accumulation of ASA and SA in synovial fluid (Figures 1 and 2). This accumulation of salicylates in joint fluid has also been observed in two studies in man [23,24] and might be explained as follows: in plasma, ASA is rapidly converted to SA via deacetylation by endogenous esterases so that SA concentrations exceed ASA concentrations shortly after administration. In the joint fluid, however, the esterase activity is much lower than in whole blood or plasma [23]. Consequently, salicylate concentrations in synovial fluid remain higher than systemic concentrations because (a) the continuous deacetylation of ASA to SA in plasma (imitated in our model by adjusting ASA and SA concentrations in the perfusion fluid) leads to a constant supply of SA into the joint and (b) ASA levels in the joint remain relatively consistent because of the lower esterase activity in synovial fluid.

To assess any anti-inflammatory effect of the salicylates measured in the synovial fluid of the *ex vivo* model, their concentrations were compared with EC_50_ values for ASA in horses. An EC_50_ value is that plasma concentration at which a half maximal effect is observed [25]. The EC_50_ values referred to were determined by Buntenkötter [26] in equine whole blood: COX-I and COX-II inhibition were assessed by the ability of ASA to inhibit clot-induced thromboxane B_2_ production or prostaglandin E_2_ production after stimulation with lipopolysaccharides, respectively.

ASA concentrations in the synovial fluid measured in the *ex vivo* model were still above the ASA EC_50_ value for COX I inhibition (0.2 μg/mL) and in the range of the EC_50_ value for COX II inhibition (2.2 μg/mL) after the systemic concentration in the perfusion fluid was below the plasma threshold of 6.5 μg/mL formulated by the international equestrian federation FEI (Figure 1).

According to our findings, it might be conceivable to abusively treat a horse with ASA in order to benefit from its short half-life and concurrent accumulation in synovial fluid even though this would clearly contradict the genuine rationale for SA thresholds, which was avoiding positive cases solely due to the horse’s diet.

## Conclusions

A threshold level for SA in plasma and urine was formulated by horse sport organisations to avoid positive cases due to the horse’s diet. Data obtained from our *ex vivo* study investigating the distribution of salicylates into joint fluid after systemic administration suggest that even with systemic concentrations below the threshold, concentrations in the synovial fluid are in the range of the EC_50_ value and may therefore be capable of exerting an anti-inflammatory effect. These results originate from an *ex vivo* setup; nevertheless, they do provide a basis to further investigate the synovial distribution of systemically administered salicylates.

## Abbreviations

ASA: Acetylsalicylic acid; SA: Salicylic acid; EC50: half maximal effective concentration; NSAID: Nonsteroidal anti-inflammatory drug; PBS: Phosphate buffered saline; RR: Relative recovery; HPLC: High performance liquid chromatography; LDH: Lactate dehydrogenase; S.E.M.: Standard error of the mean; FEI: Fédération Equestre Internationale, international equestrian federation

## Competing interests

The authors declare that they have no competing interests.

## Authors’ contributions

MF participated in the design of the study, performed the experiments and drafted the manuscript. SS participated in the design of the microdialysis experiments. JS participated in the analytical phase of the experiment. MK conceived and coordinated the study and critically revised the manuscript. All authors read and approved the final manuscript.
